# Early Determinants of Childhood Asthma in Children With Atopic Eczema and High Allergy Risk

**DOI:** 10.1111/cea.70398

**Published:** 2026-07-21

**Authors:** Reetta Puisto, Helena Ollila, Samuli Rautava, Erika Isolauri

**Affiliations:** ^1^ Department of Pediatrics, Faculty of Medicine University of Turku Turku Finland; ^2^ Department of Biostatistics University of Turku and Turku University Hospital Turku Finland; ^3^ Department of Pediatrics and Adolescent Medicine Turku University Hospital Turku Finland

## Abstract

- Most children with atopic eczema are spared from the development of asthma.
- Early life exposures can modulate children's asthma risk.

To the Editor,

1

The risk of asthma is increased in children with atopic eczema (AE) [1]. It is not fully understood why some children develop this comorbidity while others do not. The present study aimed to identify the earliest determinants of asthma in children who manifest early AE and have genetic susceptibility to atopic diseases. We hypothesized that the development of asthma is determined by modern lifestyle and healthcare‐related factors.

We combined data from three clinical probiotic intervention trials [2, 3, 4]. After including only children who had atopic mothers, had participated in the follow‐up study [5], developed AE by 2 years of age and were not twins, the final study population comprised 104 children. Informed consent was obtained from the caregivers before entering the original studies, which were all approved by the local Ethics Committee. AE was diagnosed by the study doctors at scheduled control visits during the 24‐month clinical follow‐up. Childhood asthma was determined using two distinct measures: (1) family reported asthma diagnosis during the follow‐up [5] and (2) information on recurrent purchases of inhaled corticosteroids (ICSs) from the national register by the end of 2014. Essential information on the mothers, children and childbirth was prospectively recorded.

The initial analyses used two‐sample *t*‐test, Wilcoxon rank‐sum test, chi‐squared test and Fisher's exact test to evaluate potential associations between several early life exposures and asthma development. Benjamini & Hochberg false discovery rate corrected *p*‐values were calculated, and odds ratios were evaluated. All potential associations were reanalysed using logistic regression with adjustment for follow‐up time. The impact of confounding factors was investigated using two multivariable logistic regression models based on statistical model selection which included exposures that the initial analyses suggested as potentially relevant for asthma development. In model one, asthma diagnosis was the response variable and maternal asthma, allergic sensitization by 24 months of age and age‐ and sex‐adjusted BMI (ISO‐BMI) at 24 months of age served as the explanatory variables. In model two, the response variable was the purchases of ICSs, and the explanatory variables included maternal asthma, antibiotic purchases by 6 months of age and allergic sensitization by 24 months of age. For a more precise description of the study population, methods and results, please see the Supporting Information at Zenodo.

In total, 22 of 102 children with AE were diagnosed with asthma, and 28 of 104 children had purchased ICSs. Compared to children who were spared from asthma, children with an asthma diagnosis were more frequently males, had more often mothers with asthma, purchased more frequently antibiotics during the first 6 months of life, developed more frequently allergic sensitizations and had higher ISO‐BMI at 24 months of age. The results were largely similar between children with and without recurrent ICS purchases.

The logistic regression model adjusted for the follow‐up period changed the association between recurrent purchases of ICSs and ISO‐BMI at 2 years of age from non‐significant into significant (OR 1.14, 95% CI 1.01–1.30, *p* = 0.037). No other initial results were significantly changed.

When the associations between asthma diagnosis and allergic sensitization by 2 years of age, maternal asthma and ISO‐BMI at 2 years of age were investigated in the same model, all associations remained statistically significant (Table 1). Similarly, when the associations between purchases of ICSs and allergic sensitization by 2 years of age, maternal asthma and antibiotics during the first 6 months of life were studied in the same model, all associations were statistically significant (Table 1).

**TABLE 1:** Independent associations between early life exposures and asthma in today's children with atopic eczema in two multivariable logistic regression models.

Model 1: Multivariable logistic regression analysis of asthma diagnosis with maternal asthma, early allergic sensitization and ISO‐BMI as explanatory factors				
	Children without asthma diagnosis *n* = 80	Children with asthma diagnosis *n* = 22	OR (95% CI)	*p*
Maternal asthma	12 (15.2)	8 (36.4)	3.94 (1.21–12.80)	0.023
Allergic sensitization by 2 years of age	33 (41.3)	16 (72.7)	4.09 (1.33–12.59)	0.014
ISO‐BMI at 2 years of age	22.1 (15.4–30.7)	24.2 (17.9–33.2)	1.17 (1.00–1.36)	0.045

Model 2: Multivariable logistic regression analysis of need for purchase inhaled corticosteroids with maternal asthma, early allergic sensitization and antibiotic purchases as explanatory factors				
	Children without ICS purchases, *n* = 76	Children with ICS purchases, *n* = 28	OR (95% CI)	*p*
Maternal asthma	10 (13.3)	10 (35.7)	4.43 (1.36–14.43)	0.013
Allergic sensitization by 2 years of age	31 (40.8)	19 (67.9)	5.02 (1.68–15.02)	0.0039
Antibiotic purchases by 6 months of age	4 (5.3)	6 (21.4)	8.67 (1.70–44.22)	0.0094

*Note:* Maternal asthma, allergic sensitization by 2 years of age and antibiotic purchases by 6 months of age are presented as number and percentages, whereas ISO‐BMI at 2 years of age is presented as mean and range.

Abbreviations: 95% CI, 95% Confidence Interval; ICS, inhaled corticosteroids; ISO‐BMI, age‐ and sex‐adjusted body mass index; OR, odds ratio.

This study showed that more than one in five high‐allergy‐risk children who developed AE by 2 years of age also developed asthma during childhood and adolescence. The risk of developing asthma was associated with maternal asthma, male sex, antibiotic treatment in early infancy, allergic sensitization and ISO‐BMI at 2 years of age.

Our results are consistent with both genetics and specific exposures from the modern growth environment contributing to asthma development. In accordance with contemporary microbial hypotheses, the development of asthma has been associated with delayed gut microbiota development, particularly in children with asthmatic mothers [6]. Interestingly, co‐occurrence with allergic sensitization is associated with an even more obvious microbial shift [6]. Given the role of gut microbiota in immune and epithelial barrier maturation [7], it is plausible that microbiota‐modifying exposures, including early antibiotic treatment or diet [8], might modify the risk of asthma. Overweight has also been associated with microbiota alterations, and metabolic disturbances and immune dysregulation may co‐exist [9].

It is possible that our study with a relatively modest number of subjects did not detect all exposures related to asthma risk. In addition, no claims can be made that all exposures preceded asthma development. When potential risk factors were analysed in the same model, the initial results were confirmed, suggesting that the reported associations were genuine.

Both AE and asthma are complex diseases resulting from a combination of genetic and early environmental factors. Asthma development is common in children with early AE and hereditary predisposition. It appears that the roots of the comorbidity may be found in early life, where asthma prevention strategies should also be targeted. In the future, extinguishing maternal asthma, preventing early allergic sensitization, a prudent approach to antibiotic treatment and avoidance of overweight status could help fight asthma in children.

## Author Contributions

**Reetta Puisto:** writing the original draft, formal analysis, data curation. **Helena Ollila:** formal analysis, editing the manuscript. **Samuli Rautava:** supervision, conceptualization, reviewing and editing the manuscript. **Erika Isolauri:** supervision, conceptualization, reviewing and editing the manuscript. All authors approved the final version of the manuscript.

## Funding

This work was supported by The Finnish Medical Foundation (R.P.), The Paediatric Research Foundation (R.P.), The Alfred Kordelin Foundation (R.P.), Turku University Foundation (R.P.).

## Conflicts of Interest

The authors declare no conflicts of interest.

## Data Availability

The data of this study are available upon reasonable request from the authors. For a more precise description of the study population, methods, and results, please see the Supporting Information at https://doi.org/10.5281/zenodo.19529131, which will be publicly available upon acceptance.
